# Entrectinib in the neoadjuvant setting of anaplastic thyroid cancer: a case report

**DOI:** 10.1530/ETJ-22-0179

**Published:** 2022-12-22

**Authors:** Inês Damásio, Joana Simões-Pereira, Sara Donato, Mariana Horta, Branca Maria Cavaco, Miguel Rito, Pedro Gomes, Valeriano Leite

**Affiliations:** 1 Endocrinology Department, Instituto Português de Oncologia de Lisboa Francisco Gentil, Lisbon, Portugal; 2 Nova Medical School, Lisbon, Portugal; 3 Radiology Department, Instituto Português de Oncologia de Lisboa Francisco Gentil, Lisbon Portugal; 4 Molecular Pathobiology Research Unit (UIPM), Instituto Português de Oncologia de Lisboa Francisco Gentil, Lisbon, Portugal; 5 Pathology Department, Instituto Português de Oncologia de Lisboa Francisco Gentil, Lisbon, Portugal; 6 Head and Neck Surgery Department, Instituto Português de Oncologia de Lisboa Francisco Gentil, Lisbon, Portugal

**Keywords:** anaplastic thyroid carcinoma, entrectinib, neoadjuvancy, precision medicine

## Abstract

**Background:**

Anaplastic thyroid carcinoma (ATC) is one of the most aggressive solid tumors. ATC is frequently diagnosed at advanced stages with unresectable disease and palliative care is often indicated. Recently, several patient-tailored therapies for ATC are emerging due to advances in molecular profiling of these tumors. Entrectinib is a potent oral selective inhibitor of neutrotrophic tropomyosin receptor kinase (*NTRK*), *ROS1*, and anaplastic lymphoma kinase fusions. The experience regarding ATC and other thyroid carcinomas, particularly in the neoadjuvant setting, is minimal.

**Case report:**

We present a case of a 51-year-old female patient presenting with a bulky mass of the left thyroid lobe measuring 100 × 108 × 80 mm that was considered surgically unresectable. While waiting for next-generation sequence (NGS) profiling, lenvatinib was initiated. There was an initial clinical and imagiologic response; however, progression occurred after 12 weeks, and at this time NGS identified an *ETV6-NTRK3* fusion and entrectinib was started. After 12 weeks, tumor diameters reduced to a minimum of 68×60×49 mm, and the patient underwent total thyroidectomy plus central lymphadenectomy. Histological diagnosis confirmed an ATC (pT4a R2 N1a). Adjuvant radiotherapy (RT) (60 Grays) with weekly paclitaxel (45 mg/m^2^) was then administered followed by maintenance entrectinib 600 mg daily. Fluorodeoxyglucose positron emission tomography performed 3 months after completion of RT showed only non-specific uptake in the posterior wall of the hypopharynx and larynx, suggestive of inflammation.

**Conclusion:**

We report the first case of an ATC with a dramatic response to neoadjuvant therapy with entrectinib, which enabled surgical resection of an *ab initio* unresectable tumor.

**Established facts**

- Anaplastic thyroid carcinoma is one of the most aggressive cancers in humans and frequently presents with unressectable disease.
- Individualized therapies targeting specific molecular alterations are emerging.

**Novel insights**

- We present the first reported case of a patient with an *NTRK*-positive ATC showing a successful response to neoadjuvant treatment with entrectinib.

## Introduction

Anaplastic thyroid carcinoma (ATC) accounts for 1–2% of all thyroid malignancies. Despite its rarity, ATC is one of the most aggressive solid tumors in humans, with a median survival of 5–6 months (1).

Historically, the standard treatment for ATC includes surgical resection, if feasible, followed by radiotherapy (RT) and radiosensitizing chemotherapy (CTX). Unfortunately, most patients with ATC are not surgical candidates due to unresectable disease at diagnosis. In these cases, RT or CTX can be performed for local or systemic control, respectively. However, results of these therapies are usually disappointing, turning palliative care as the only reasonable approach in many cases (2). Recently, comprehensive investigation of the genomic and transcriptomic landscape of ATC has raised new hope for clinicians and patients, by providing specific therapeutic targets that may allow for individualized therapy.

Gene fusions involving the neutrotrophic tropomyosin receptor kinase (*NTRK*) gene family – *NTRK1*, *NTRK2*, and *NTRK3* – lead to oncogenesis by generating chimeric proteins with a constitutively activated kinase function, which stimulate cellular proliferation via the RAS/RAF/MAPK pathway (3).

*NTRK* fusions are rarely found in solid tumors, including adult thyroid carcinomas. However, they may represent a molecular target for cancer therapy, and the current American Thyroid Association (ATA) (4) ATC guidelines recommend analysis of somatic fusions in tumors without other candidate driver genes, such as BRAF.

Currently, there are two drugs, approved by Food and Drug Administration (FDA) and by the European Medicines Agency (EMA), targeting *NTRK* fusions – larotrectinib and entrectinib – which represent two of the few ‘tumor-agnostic’ treatments available. Larotrectinib, a selective pan-TRK inhibitor, was evaluated in TRK fusion-positive thyroid cancers in three different clinical trials (NCT02122913 (5), NCT02637687 (6), and NCT02576431 (https://clinicaltrials.gov/ct2/show/NCT02576431)). A pooled analysis of two of these trials (NCT02122913 and NCT02576431) included 28 patients with locally advanced or metastatic thyroid carcinoma, of which 19 patients had papillary thyroid carcinoma (PTC), seven patients had ATC, and two patients had follicular carcinoma. An objective response was observed in three patients with ATC (two had a partial response and one had stable disease) (7).

Entrectinib is a selective inhibitor of TRK ROS1 and anaplastic lymphoma kinase fusions. An integrated analysis of three ongoing early-phase trials (8) with patients with metastatic or locally advanced solid tumors harboring oncogenic *NTRK1 NTRK2* and *NTRK3* gene fusions treated with entrectinib included only five patients with thyroid carcinoma, and histological characteristics and response were not available in the report. Both drugs had a favorable safety profile and adverse events were primarily grades 1 and 2.

Herein, we report the first case of an ATC with a dramatic response to neoadjuvant therapy with entrectinib, which enabled surgical resection of an *ab initio* unresectable tumor.

## Case report

We report a case of a 51-year-old female patient, with a medical history of type 2 diabetes mellitus, obesity, and asthma, evaluated for the first time in our center in May 2021. She mentioned a rapidly growing cervical mass and dyspnea since January 2021, when a neck ultrasound documented a hypoechogenic and heterogeneous solid nodule on the left thyroid lobe with 49 × 35 × 63 mm; fine needle biopsy was non-diagnostic.

**Figure 1:**
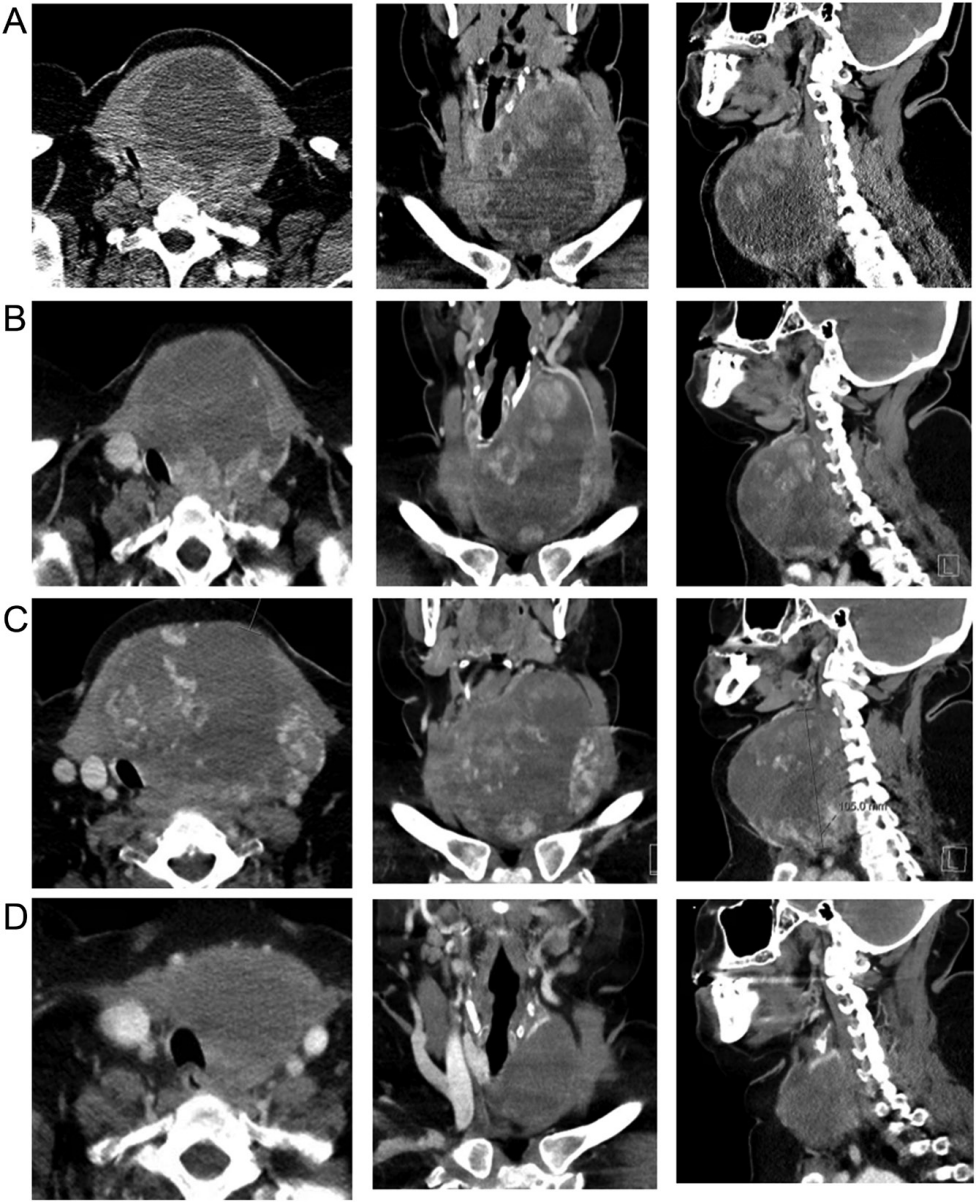
Neck CT scans showing the effect of lenvatinib and entrectinib on tumor size. (A) Before lenvatinib – May 2021. (B) After 2 weeks of lenvatinib – June 2021. (C) After 12 weeks of lenvatinib – August 2021. (D) After 9 weeks of entrectinib – October 2021.

**Figure 2:**
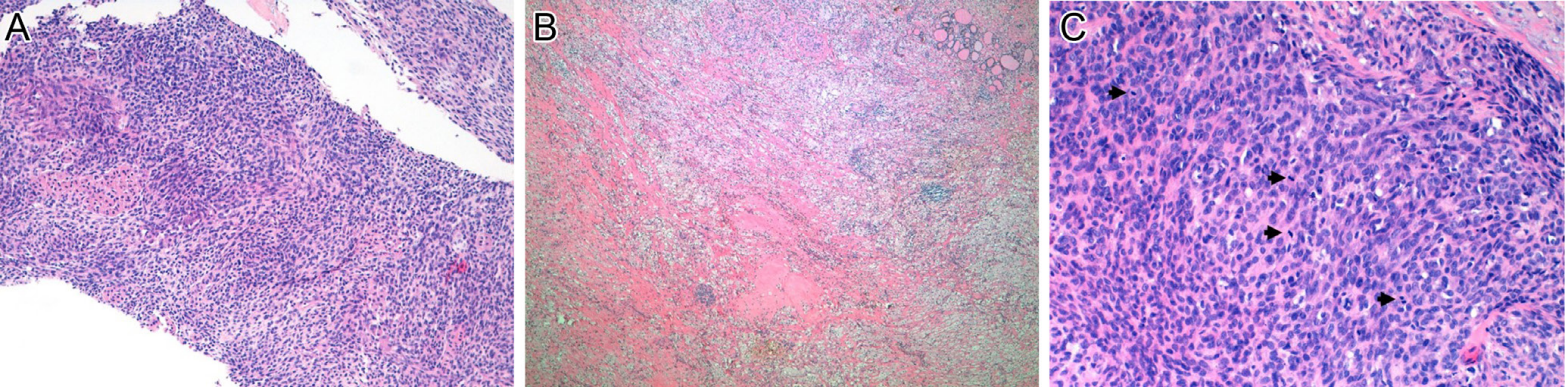
Biopsy sample and surgical specimen. (A) (H&E stain): Biopsy sample. Fragments of a hypercellular, spindle cell, undifferentiated neoplasm. Scattered islands of cells with more vast eosinophilic cytoplasm could be seen. There was mitotic activity, sometimes with atypical mitosis. The cells were focally positive for PAX8 and TTF1 (data not shown). (B) (H&E stain): Surgical specimen. Most of the thyroid gland was replaced by fibrosis, necrotic tissue, and chronic inflammatory infiltrate with foamy macrophages, lymphocytes, and giant multinucleated cells, probably areas where, previously, the anaplastic carcinoma was present. On the upper right corner, residual normal thyroid tissue can be seen. (C) (H&E stain): Surgical specimen. Still, residual foci of the undifferentiated neoplasm similar to those observed in the previous biopsy could be seen. In this field, several mitotic figures can be identified (arrowheads).

Neck and chest computed tomography (CT) performed in our center (May 2021) showed a bulky mass of the left thyroid lobe measuring 100 × 108 × 80 mm (volume of 449.3 cm^3^), with hypodense necrotic areas, causing deviation and narrowing of tracheal lumen to a minimum area of 30 mm (Fig. 1A). There was also deviation of the primitive carotid artery and of the jugular vein without apparent invasion of their walls. There were no lymph nodes or lung metastases. Fluorodeoxyglucose positron emission tomography (FDG-PET) showed significant uptake by the tumor (SUV_max_ = 24.4) without uptake in other tissues. The tumor was considered unresectable at this point. A core biopsy was performed and histopathological evaluation was compatible with ATC (Fig. 2A). Thyroid function tests were normal, serum thyroglobulin was 35.2 ng/mL (normal range <55), and serum calcitonin was <2.0 pg/mL (normal range <8.5). *BRAF* was wild type by Idylla™ NRAS-BRAF-EGFR S492R Mutation assay (Biocartis, Mechelen, Belgium), a real-time polymerase chain reaction-based molecular testing system. While waiting for the results of the next-generation sequencing (NGS) analysis of a multigene panel, the patient was put on systemic therapy with lenvatinib 24 mg daily. After 2 weeks, there was improvement of the dyspnea, and the neck CT showed stable disease (94 × 98 × 72 mm; volume of 344 cm^3^), but tracheal lumen area increased to 126 mm (Fig. 1B). NGS analysis using the Ampliseq^TM^ for Illumina Focus Panel (Illumina, San Diego, CA, USA) identified an *ETV6–NTRK3* fusion. Given the stability of the disease and some institutional constraints in obtaining an *NTRK* inhibitor, the patient was maintained on lenvatinib. She had mild-to-moderate adverse effects (hypertension, mucositis, diarrhea, and hypothyroidism), and the dosage of 24 mg daily was maintained.

After 12 weeks on lenvatinib, neck CT showed disease progression with an increase of the mass to 110 × 102 × 81 mm (volume of 473 cm^3^), as shown in Fig. 1C. Tracheal and cervical vessel compression was more evident. The left primitive carotid artery was surrounded by the tumor in an angle of 90–180°. The esophagus was also deviated, and there was no cleavage plan with the tumor. Lenvatinib was then switched to entrectinib 600 mg daily (August 2021). Two days later, the patient presented with dysphagia, dysphonia, and increase of the cervical mass. At this point, necrosis-related inflammation due to entrectinib was hypothesized and the patient was hospitalized and started a high-dose corticoid regimen (dexamethasone 4 mg per 6 h). There was a visible reduction in tumor size and clinical improvement in the following week, and the patient was discharged. Corticoids were then slowly tapered down. No more entrectinib-associated adverse events were observed, except mild fatigue and muscle pain in the legs.

The patient remained clinically stable, and a neck CT performed after 6 weeks while on entrectinib showed a marked reduction of the tumor to 78 × 67 × 52 mm (volume of 141.3 cm^3^), with improvement of tracheal compression and with no signs of invasion of the carotid artery or of the pre-vertebral muscles. CT was repeated 3 weeks later and showed further reduction of tumor dimensions to 68 × 60 × 49 mm (volume of 104.3 cm^3^), with no invasion of adjacent structures (Fig. 1D). At this point, ATC was considered surgically resectable, and the patient underwent total thyroidectomy plus central lymphadenectomy. Histological analysis (Fig. 2B and C) revealed the presence of residual foci of ATC with tracheal invasion, along with alterations related to therapy, two foci of papillary microcarcinomas with 6 and 9 mm in the right thyroid lobe, and a metastatic perithyroid lymph node measuring 2 mm (pT4a R2 pN1a).

Therapy with entrectinib was resumed after surgery and suspended just before the initiation of adjuvant RT (60 Grays) with weekly paclitaxel (45 mg/m^2^) which she completed on February 18, 2022.

She resumed therapy with entrectinib 600 mg/day on March 15, 2022, with reasonable tolerability but had to discontinue the drug for 3 days and reduce the dose to 400 mg/day in April due to arthralgias and myalgias.

FDG-PET in June 2022 showed a non-specific uptake in the posterior wall of the hypopharynx and larynx, suggestive of postradiation inflammation, which was confirmed by a neck and chest CT scan performed in September 2022.

## Discussion

ATC is one of the most lethal carcinomas and is generally refractory to standard therapies (9). The knowledge of the genomic landscape of ATC has become extremely important since it allows the use of new drugs targeting specific molecular alterations (1). Among the actionable mutations that occur in ATC, the most common is *BRAF* p.V600E (50–70%) (4) Combined therapy with the *BRAF* inhibitor dabrafenib and the *MEK* inhibitor trametinib was approved by the FDA in 2018 for ATC harboring *BRAF* p.V600E mutations, either in stage IVC or in IVB (2, 4). In the absence of *BRAF* p.V600E mutations, ATA guidelines suggest to search for other molecular targets such as *NTRK* or RET/PTC fusions (4).

*NTRK* rearrangements occur in approximately 0.3% of all solid tumors and have a prevalence of around 2.3% in thyroid carcinomas (10, 11), being more frequent in pediatric PTC and in PTC occurring after the Chernobyl radioactive fallout. In a series of 11 cases from a single institution, *TRK* fusion-positive thyroid carcinomas were clinically aggressive with a high metastatic rate, multinodular growth, and lymphovascular spread (3). Xu Bi *et al.* (12) recently reported, after the molecular analysis of 107 ATCs, *NTRK* fusions in two ATC patients: one harboring *NTRK1–IRF2BP2* and the other *NTRK2–CRNDE*. Park *et al.* (13) described four patients with advanced thyroid cancer harboring *NTRK* gene fusions, treated with larotrectinib: three patients with PTC or poorly differentiated thyroid carinoma (PDTC) achieved durable radiographic responses. The patient with ATC started larotrectinib after total thyroidectomy, and a 16% reduction in tumor burden after 2 months was reported. However, after 6 months, disease progressed.

The use of lenvatinib has been approved for ATC treatment in Japan. However, a phase 2 trial of lenvatinib in ATC, through the International Thyroid Oncology Group, was suspended due to lack of efficacy, so its use is not recommended for ATC in the most recent ATA guidelines (4). Iñiguez-Ariza *et al.* (14) conducted a retrospective study of lenvatinib in 28 ATC patients and reported transient benefits and important toxicities. Barbaro *et al.* described two cases of ATC with a rapid response to lenvatinib even at a low dose (14 mg). In one of the cases, lenvatinib was used in the neoadjuvant setting (9). In our patient, lenvatinib was used transiently while awaiting for the results of molecular analysis, with a rapid initial response and disease stability for some weeks.

In sum, this case emphasizes the importance of identifying actionable mutations, even for neoadjuvant treatment, since targeted therapy can dramatically change the course of ATC. As far as we know, this is the first report of the use of entrectinib in a patient with an ATC harboring an *NTRK* fusion in the neoadjuvant setting.

## Declaration of interest

The authors declare that there is no conflict of interest that could be perceived as prejudicing the impartiality of this case report.

## Funding

This work was supported by Instituto Português de Oncologia de Lisboa Francisco Gentil, EPE (IPOLFG).

## Statement of ethics

The patient provided written informed consent for this case report.

## Author contribution statement

Every person listed as authors made significant contribution to the writing and revision of this case and approved the final version for submission for publication.

## Acknowledgement

The authors thank Dr Joana Maciel for her help in taking care of the patient when entrectinib was started and Dr Ana Saramago for performing genetic testing.
